# Transient dynamics of Aβ contribute to toxicity in Alzheimer’s disease

**DOI:** 10.1007/s00018-014-1634-z

**Published:** 2014-05-07

**Authors:** E. Hubin, N. A. J. van Nuland, K. Broersen, K. Pauwels

**Affiliations:** 1 Nanobiophysics Group, MIRA Institute for Biomedical Technology and Technical Medicine, Faculty of Science and Technology, University of Twente, 7500 AE Enschede, The Netherlands; 2 Structural Biology Brussels, Department of Biotechnology (DBIT), Vrije Universiteit Brussel (VUB), Pleinlaan 2, 1050 Brussels, Belgium; 3 Structural Biology Research Center, VIB, Pleinlaan 2, 1050 Brussels, Belgium

**Keywords:** Alzheimer’s disease, Amyloid-β peptide, Aβ dynamics, Intrinsically disordered peptide, Aggregation

## Abstract

The aggregation and deposition of the amyloid-β peptide (Aβ) in the brain has been linked with neuronal death, which progresses in the diagnostic and pathological signs of Alzheimer’s disease (AD). The transition of an unstructured monomeric peptide into self-assembled and more structured aggregates is the crucial conversion from what appears to be a harmless polypeptide into a malignant form that causes synaptotoxicity and neuronal cell death. Despite efforts to identify the toxic form of Aβ, the development of effective treatments for AD is still limited by the highly transient and dynamic nature of interconverting forms of Aβ. The variability within the in vivo “pool” of different Aβ peptides is another complicating factor. Here we review the dynamical interplay between various components that influence the heterogeneous Aβ system, from intramolecular Aβ flexibility to intermolecular dynamics between various Aβ alloforms and external factors. The complex dynamics of Aβ contributes to the causative role of Aβ in the pathogenesis of AD.

## Introduction

**Fig. 1:**
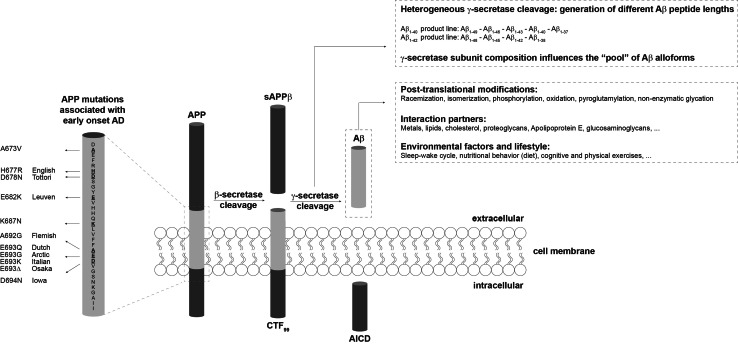
Heterogeneity in the Aβ peptide pool. Sequential proteolytic events by the β- and γ-secretase of the amyloid precursor protein (APP) give rise to the carboxy-terminal fragment (CTF), APP intracellular domain (AICD), and the amyloid-β peptide (Aβ). The heterogeneity in the Aβ pool originates from the proteolysis by the γ-secretase, but also post-translational modifications contribute to the formation of various Aβ alloforms. Mutations in Aβ and other exogenous factors can influence the dynamics that are observed within the Aβ system

Experimental studies and clinical trials are ongoing in the search for an effective prevention or treatment of Alzheimer’s disease (AD) [1–3]. These studies and trials often target the amyloid-beta peptide (Aβ), which plays a major role in AD pathogenesis [4]. Effective drug development has remained without success and this is thought to originate from the fact that Aβ can appear in many different shapes that can interconvert within a dynamical interplay. This finding triggered a vast exploration of the many conformations the peptide can adopt, as well as the aim to precisely pinpoint which of these conformations can be claimed as “the toxic species”, such that specific drug targeting can be employed. To complicate matters even more, a heterogeneous pool of monomeric Aβ varying in length from 37 to 49 amino acids is produced by proteolytic cleavage from the transmembrane amyloid precursor protein (APP) by β- and γ-secretases [5, 6] (Fig. 1). Most research effort has been focused on the most abundant form Aβ_1−40_, which comprises 40 amino acids. The longer and less abundant Aβ_1−42,_ C-terminally extended by two residues, has been found to be more aggregation-prone [7]. Nonetheless, it has recently been discovered by us [8–10] and other groups [11–14] that the co-occurrence of peptides varying in length can affect the neurotoxic and aggregation potential of the total Aβ pool. It was also recognized that particularly small aggregated forms of Aβ are potently toxic, rather than the mature amyloid fibrils as observed in the brain of AD patients. Therefore, a lot of research has aimed at understanding the Aβ aggregation mechanism and identifying the intermediate species that occur along the aggregation pathway [15, 16]. The current amyloid cascade hypothesis suggests that AD-related synapto- and neurotoxicity might be mediated by soluble Aβ oligomers [17, 18], which have proven notoriously difficult to study in detail in vivo with the currently available technology. The dynamics, stability, and transient lifetime of potentially toxic species further hamper the possibility to precisely pinpoint the toxic structural aspects of Aβ aggregates. Moreover, the dynamic behavior of aggregation intermediates may actually provide an important source for toxicity of Aβ as isolated Aβ oligomers are only toxic in the presence of Aβ monomers that provide a source for continued growth of oligomers into fibrillar species [13, 19].

This review discusses how Aβ peptide dynamics can influence and contribute to Aβ-induced toxicity. Aβ dynamics is mainly considered on two levels. First, we define *intramolecular dynamics* of Aβ as the intrinsic disorder or polypeptide backbone flexibility that is present in isolated Aβ monomeric peptides or aggregation states. Second, we define *intermolecular dynamics* as (1) the interplay between different Aβ alloforms present in the in vivo Aβ pool and (2) the dynamical equilibrium that exists between different Aβ species. With the term alloform, we refer to a distinct form of the Aβ peptide that is commonly treated as a single kind of peptide species, like Aβ length variants or side chain modifications. Finally, several external factors and interaction partners that can influence Aβ dynamics are addressed. The potential importance of Aβ dynamics in understanding AD pathology is highlighted with the aim of shaping new research orientations for AD treatment.

## Intramolecular dynamics

The Aβ monomer has a high tendency to self-assemble into large aggregates and fibrils. It is increasingly recognized that despite the highly packed and ordered state of these higher-order aggregates, they often do contain a significant portion of flexible and intrinsically disordered regions [20]. The intrinsically disordered nature of the Aβ monomer is fairly well documented, but revealing the structural disorder in oligomers and fibrils has proven more challenging due to the difficulties in studying this phenomenon. In this section, we discuss the intrinsic structural disorder that is present in every Aβ aggregation state, and we illustrate how it contributes to Aβ-induced toxicity.

### The intrinsically disordered Aβ monomer

**Fig. 2:**
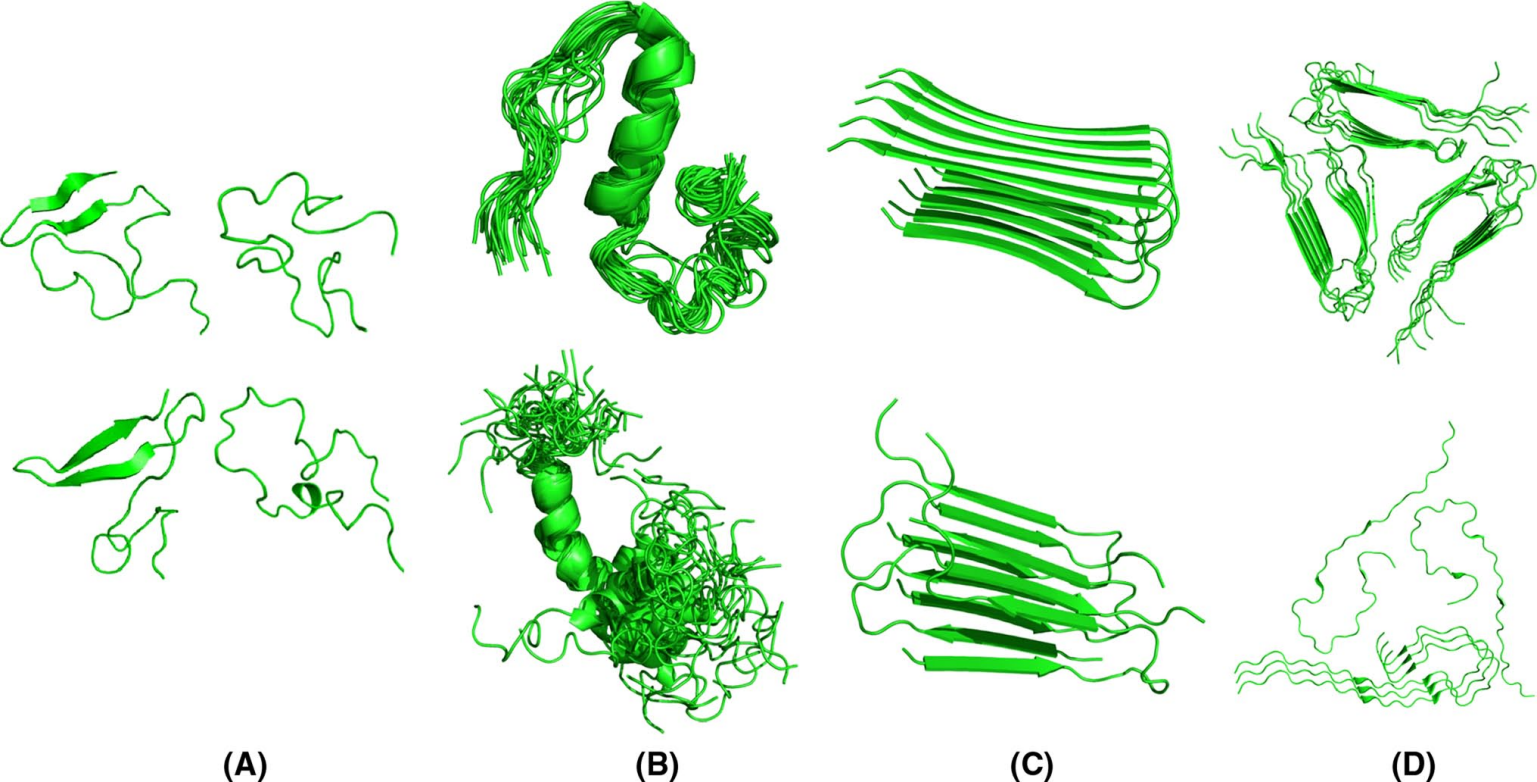
Various structures of Aβ that correspond to different experimental conditions and phases in the aggregation landscape. **a** Four representatives of the structural ensemble of monomeric Aβ_1−42_ under aqueous conditions as derived from a combined molecular dynamics/NMR approach [38]. Extended as well as collapsed coil conformations with secondary structural elements can be observed. **b** Aβ_1−40_ in presence of 50 mM NaCl at 15 °C [33] and Aβ_1−42_ in presence of 30 % hexafluoroisopropanol [32] contain an α-helical segment. **c** Fibril polymorphism illustrated by fibrillar Aβ_1−42_ [53], D23N Aβ_1−40_ [74] and **d** the ultrastructure of Aβ_1−40_ [83], and brain-derived Aβ_1−40_ [89]

Although the pathological hallmark of AD comprises insoluble Aβ deposits in neuritic plaques in the brain of AD patients, monomeric Aβ peptides have also been purified and characterized from brain tissue [21–24]. Size exclusion chromatography (SEC) experiments suggested that the freshly dissolved peptide eluted as a single low molecular weight species, consistent with a monomer or dimer [25–27]. These low molecular weight Aβ species were competent to deposit onto pre-existing amyloid in preparations of AD cortex, with a first-order kinetic dependence on soluble Aβ concentration [26]. Translational diffusion measurements by nuclear magnetic resonance (NMR) techniques conclusively demonstrated that the form of the peptide active in plaque deposition is a monomer [26]. Further NMR data revealed that monomeric Aβ exists in solution as disordered coils that lack regular α-helical or β-stranded structure [28–30]. Despite the challenging task because of its unstructured and amyloidogenic nature, the Aβ monomer is now well recognized as an intrinsically disordered peptide (IDP). This implies that the monomeric Aβ peptide does not display a unique fold, as would be the case for a typical well-folded protein, but rather comprises a mixture of rapidly interconverting conformations whereby the polypeptide backbone can sample the conformational space without any stable and well-defined conformational ensemble (Fig. 2). Yet, it is possible to bias the ensemble toward distinct secondary structure elements by changing solution conditions and/or the oxidation state of Met_35_ [30–33].

Some experimental studies suggested that Aβ is not entirely a “random coil”. Ion mobility mass spectrometry (MS) combined with theoretical modeling showed that Aβ_1−42_ in aqueous solution adopts both extended chain as well as collapsed-coil structures [34]. Limited proteolysis successfully identified structured and disordered regions within Aβ [35]. This approach revealed a proteolytically resistant decapeptide, Ala_21_–Ala_30_, that was found in NMR studies to form a turn-like structure [30]. When the dynamics of monomeric Aβ_1−40_ in solution was studied using ^15^N-relaxation experiments, it revealed structural propensities that correlate well with the secondary structure segments of the peptide that are present in the fibrils, and with the α-helical structure in membrane-mimicking systems [32, 36]. NMR studies further revealed subtle differences between Aβ_1−40_ and Aβ_1−42_ monomers whereby a modest increase in C-terminal rigidity has been observed in Aβ_1−42_ versus Aβ_1−40_ [37]. Various molecular dynamics simulations also hinted that distinct intramolecular interaction patterns occur in Aβ_1−42_ [28, 38, 39]. Such subtle differences between Aβ_1−40_ and Aβ_1−42_ were confirmed by molecular dynamics simulations [40, 41]. Experimental results in combination with computational simulations have thus proven very powerful to shed light on the conformational landscape of IDPs. The emerging picture of Aβ comprises an IDP that can adapt a variety of collapsed and extended monomeric conformations and transiently samples long-range intramolecular interactions without exclusively stabilizing a specific globular fold.

Even though the physiological function of Aβ remains obscure, the intrinsic structural flexibility offers certain advantages: high specificity and low affinity in the case of binding-induced folding IDPs (mostly exploited in signaling pathways), and high binding promiscuity that is frequently used by hub proteins in large interaction networks [42]. So its IDP nature facilitates the interaction of the peptide with many different binding partners (see “Other players in the game”), including identical peptides and other Aβ alloforms. In addition, the high intramolecular flexibility of Aβ also simplifies post-translational modifications because the involved side chains are readily accessible (see “The in vivo Aβ pool: a cocktail of different interacting species”).

There is a well-established link between intrinsic polypeptide disorder and functional promiscuity. Protein moonlighting, the phenomenon of proteins exhibiting more than one unique biological function, is typically mediated by intrinsically disordered regions in polypeptides [43]. As IDPs can play a role in numerous biological processes, it is not surprising to find some of them involved in human diseases.

### Intrinsic fibril flexibility can underlie disease progression and phenotype

Aβ fibrils contain high order and rigidity compared to Aβ monomers, but still retain a considerable amount of disorder in the N-terminal segment [44–47] and they are often polymorphous. The inherent disorder of Aβ fibrils and the associated fibril polymorphism could underlie time-dependent structural changes during aging in AD and differences in disease progression and phenotype.

#### The molecular dynamic nature of Aβ fibrils

Even though the amyloid fibril state of Aβ has traditionally been viewed as a rigid or semi-rigid state with the typical cross-β X-ray fiber diffraction pattern [48, 49], part of the peptide in this conformation is also flexible. This flexibility has been illustrated first by solid-state and solution NMR [50–54], electron paramagnetic resonance (EPR) [44], site-directed mutagenesis [55], limited proteolysis and hydrogen-deuterium exchange (HDX) evaluated by MS [45–47, 56], and even X-ray crystallography [57]. These studies suggested a hairpin-like arrangement of each Aβ monomer stacked within the fibril, consisting of two semi-rigidly organized β-strands linked by a flexible connecting region (Fig. 2). The hydrophobic C-terminus of Aβ_1−42_ in the fibril is highly resistant to HDX and forms the fibril core [53, 54]. In contrast, the C-terminus of Aβ_1−40_ in the fibril contains slightly more disorder [52, 56, 58–61]. The N-terminal segment, which can range from 10 to 19 residues depending on the study, remains intrinsically disordered for both Aβ_1−40_ and Aβ_1−42_ fibrils (Table 1). This relatively hydrophilic part of the polypeptide chain is excluded from the H-bonded β-sheet fibril core and remains exposed to the solvent [44–47, 52–54, 58–61]. Recently, differential scanning calorimetry suggested that thermal denaturation of amyloid fibrils can take place and that this process can be considered as a reversible equilibrium under certain experimental conditions, highlighting the dynamic nature of fibrils [62]. These observations illustrate the impact of the various dynamics within the Aβ system.

**Table 1:** Secondary structure assignments of Aβ fibrils and structures deposited in the PDB

Peptide	Flexible regions (solvent-exposed)	β-structured regions (non-exposed)	Method	References
Aβ_1−40_	N-terminus (Asp_1_-Phe_19_) C-terminus (Met_35_-Val_40_)	Phe_20_-Leu_34_	HDX-MS coupled with online proteolysis	[191]
Aβ_1−40_	N-terminus (Asp_1_-His_14_) C-terminus (Gly_37_-Val_39_) Turn? (Ser_26_-Asn_27_)	Gln_15_-Asp_23_ Lys_28_-Met_35_	HDX-solution NMR	[52]
Aβ_1−40_	N-terminus (Asp_1_-His_14_) C-terminus (Gly_37_-Val_40_) Turns (Glu_22_-Asp_23_, Gly_29_-Ala_30_)	Gln_15_-Ala_21_ Val_24_-Lys_28_ Ile_31_-Val_36_	Scanning proline mutagenesis	[58]
Aβ_1−40_	N-terminus (Asp_1_-Tyr_10_) Bend (Gly_25_-Gly_29_)	Val_12_-Val_24_ C-terminus (Ala_30_-Val_40_)	Solid-state NMR	[50]
Aβ_1−40_	N-terminus (Asp_1_-Gly_9_) Bend/loop (Asp_23_-Gly_29_)	Tyr_10_-Glu_22_ C-terminus (Ala_30_-Val_40_)	Solid-state NMR	[59]
Aβ_1−40_ Aβ_1−42_	N-terminus (Asp_1_-Tyr_10_) C-terminus (Val_40_-Ala_42_?) Turn/bend? (Asp_23_-Gly_29_)	His_14_-Gly_38_	Site-directed spin labeling-EPR	[44]
Aβ_1−42_	N-terminus (Asp_1_-Leu_17_) Turn (Asn_27_-Ala_30_)	Val_18_-Ser_26_ C-terminus (Ile_31_-Ala_42_)	HDX-solution NMR	[53]
Aβ_1−42_	N-terminus (Asp_1_-Tyr_10_) Bend region? (Ser_26_-Asn_27_)	Glu_11_-Gly_25_ C-terminus (Lys_28_-Ala_42_)	HDX-solution NMR	[54]

Fibril structures deposited in the PDB: synthetic Aβ_1−40_ (2LMN, 2LMO, 2LMP, 2LMQ), brain-derived Aβ_1−40_ (2M4J), synthetic D23N Aβ_1−40_ (2LNQ), recombinant Aβ_1−42_ (2BEG)

The inherent flexibility of Aβ fibrils also allows the internal fibril structure to evolve in time. Multidimensional infrared spectroscopy revealed that fresh and 4-year-old fibrils were structurally heterogeneous due to trapped water molecules that perturbed the H-bonding pattern in time [63]. Recently, Nilsson and coworkers [64] revealed conformational rearrangements during aging in plaques in the brains of AD mouse models using different luminescent conjugated polythiophenes.

Although ignored for a long time, structural disorder in fibrils seems to occur in various amyloidogenic proteins (e.g. α-synuclein, tau, and multiple prions) (reviewed in [20]). Structural disorder in fibrils has been suggested to stabilize fibril formation by accommodating destabilizing residues and by limiting the unfavorable entropy associated with the formation of the highly ordered cross-β spine.

#### Aβ fibrils are polymorphic entities

Overall fibril topology has been studied using cryo-electron microscopy and 3D reconstruction. In general, Aβ fibrils exhibit multiple distinct morphologies that can differ in fibril symmetry, width, twist period, and curvature [65, 66]. This structural diversity is not limited to Aβ fibrils, but appears to be a fundamental property of the amyloid state [67–69]. *Inter*-*sample polymorphism* commonly occurs in vitro in different fibril growth conditions and is subject to pH, temperature, agitation, and salt conditions [70, 71]. A Darwinian-type “survival of the fittest” competition allows the type of fibril that is kinetically the most accessible in a given environment to be the most populated [72]. However, Aβ_1−40_ can also form at least 12 structurally distinct morphotypes under the same solution conditions (*intra*-*sample polymorphism*) indicating that this polymorphism arises from an intrinsic variability [73]. Interconversion between fibril polymorphs coexisting in solution can occur, resulting in the thermodynamically more stable polymorph, as was monitored by solid-state NMR over a period of several weeks for Aβ_1−40_ [74, 75].

Amyloid polymorphism can have several molecular origins that are not mutually exclusive [76–79]. First, mass-per-length values obtained from scanning TEM indicate that fibrils can be composed of one to five protofilaments (the minimal fibrillar entities) [80, 81]. Second, distinct orientations and modes of lateral association of protofilaments by different patterns of inter-residue interactions determine if protofilaments are oriented side-by-side [50, 82], offset from one another [76, 77], or winded around a hollow core [79]. Third, solid-state NMR demonstrated that agitated (striated) and quiescent (twisted) fibrils differ in the residues participating in the β-strands and such variations in the underlying protofilament substructure can contribute to polymorphism [59, 83]. Surprisingly, the Iowa mutant (D23N Aβ_1−40_) was recently found to form metastable fibrils with an antiparallel cross-β spine, indicating that a familial disease-related mutation can have profound effects on fibril structure [74]. Although the cross-β spine of Aβ fibers is a common feature, fibrils show a great variety of structural complexity that appears inherent to the dynamic nature of the peptide.

#### Fibril polymorphism could lead to different pathological outcomes

Fibrils can initiate inflammation in brain tissues and cell-cultured microglia and astrocytes. Fibril-induced inflammation then leads to the secretion of pro-inflammatory cytokines and the production of free radicals causing oxidative damage [84, 85]. Substantial evidence provided that different fibril morphologies exert different toxicities in vitro, although toxic activity of oligomeric Aβ was reported to exceed that of the fibrillar form multiple times [53, 59, 86–88]. For example, oligomeric Aβ correlated more strongly to cognitive impairment as compared to fibrillar Aβ of amyloid plaques [86, 87].

Fibril polymorphism could explain the weak correlation between plaque load and cognitive impairment. If plaques are comprised of different fibril polymorphs, different levels of toxicity could be associated to these amyloid deposits. In this case, the structural diversity of fibrils may account for differences in disease progression and phenotype as has been suggested by Tycko and coworkers [89]. They reported that Aβ fibrils seeded from human brain extracts differed between patients with different clinical history and neuropathology [89]. Moreover, fibril polymorphism has been linked previously to different phenotypes for hereditary transthyretin amyloidosis [90]. In this regard, the different architectures of wild-type Aβ and Iowa D23N fibrils, comprising respectively parallel and antiparallel β-sheet orientations, could underlie the different pathological outcomes: sporadic AD versus early onset AD associated with cerebral amyloid angiopathy (CAA).

### Aβ oligomers: a mishmash of conformations and sizes

Since the Aβ plaque load and AD severity could not be correlated [86, 87], growing evidence has revealed that soluble oligomers, either on- or off-pathway to fibrils (see “The in vivo Aβ pool: a cocktail of different interacting species”), play a primary role in AD. Soluble oligomers have commonly been associated with disease severity, the loss of synapses and neuronal damage (reviewed in [18]). The low abundance, heterogeneity, low solubility, and transient nature of Aβ oligomers have hindered structural studies. It now becomes clear that Aβ oligomers exist in a broad range of interconverting assemblies varying in size, conformation, and associated toxicity (reviewed in [91, 92]).

Aβ oligomers can cause toxicity by a variety of mechanisms (reviewed in [93]). To enable drug design, it is essential to establish the key determinants of oligomer toxicity. Several studies report that neurotoxic activity varies with Aβ oligomer size with small oligomers (*n* < 14) being most toxic [94, 95]. However, oligomer size is not sufficient to define toxicity as Aβ oligomers with similar size have been shown to exert different toxicities [96–98]. The underlying peptide conformation also needs to be taken into account as the interplay between Aβ oligomer size and conformation plays an important role in toxicity (reviewed in [92]). The design of a well-controlled study to investigate size and conformational impact on toxicity is notoriously difficult as different oligomer conformations and sizes are in continuous exchange. However, studies in which different conformations or sizes have been enriched or stabilized by means of crosslinking have been performed and careful conclusions can be drawn from such studies. For example, different Aβ oligomer conformations have been shown to induce neurotoxicity by distinct mechanisms in human cortical neurons [99]. One possibility to classify oligomers according to their underlying structure is based on recognition by conformation-dependent antibodies [100–103]. Surprisingly, soluble oligomers of a wide variety of amyloidogenic polypeptides (Aβ, α-synuclein, islet amyloid polypeptide, polyglutamine, lysozyme, human insulin and prion peptide) react with the oligomer-specific A11 antibody developed in the laboratory of Charles Glabe, suggesting that there has to be a common denominator to their toxic origin. Interestingly, pre-incubation of mouse hippocampal neurons with the A11 antibody, before treatment with Aβ, rescues them from the neurotoxic effects induced by Aβ [8]. It has been suggested that A11 positive oligomers are composed of antiparallel β-sheets, based on Fourier transform infrared (FTIR) spectroscopy. This antiparallel signature might represent a critical step in perturbation or permeabilization of cell membranes leading to cell toxicity [104]. Later studies using FTIR, EPR, and X-ray crystallography have confirmed that oligomeric species can be characterized by an antiparallel β-sheet orientation, while most fibrils consist of in-register, parallel β-sheets [105–109]. Moreover, antiparallel oligomers displayed a lower content in secondary structure and faster HDX kinetics compared to fibrils, suggesting a higher intrinsic flexibility [104].

Apart from size and peptide conformation, this intrinsic flexibility of the Aβ oligomer can also be a key determinant of Aβ-induced toxicity. Several studies have shown that the N-terminus retains a degree of flexibility upon oligomerization and is exposed to the solvent [41, 109–111]. Ahmed and coworkers reported solution NMR measurements of Aβ_1−42_ pentamers [111]. The authors found that the loosely packed N-terminal segment of Aβ was defined by HDX ratios approaching 1 for residues Asp_1_-Gly_9_, indicating high solvent accessibility and nearly complete exchange within the acquisition time (<1.5 h). In contrast, Val_40_-Ala_42_ were less solvent accessible and most likely buried within the center of the oligomer. Similar results were obtained for packing of the Aβ peptide within Aβ_1−42_ dodecamers. Site-directed spin labeling of Aβ_1−42_ combined with EPR spectroscopy showed that the N-terminus was loosely packed within the dodecamer, while residues Ile_32_-Val_40_ formed a tight core [109]. Increased structural disorder and solvent exposure of hydrophobic segments of the oligomer have been suggested to be a common feature of highly toxic, soluble aggregates [96–98, 112]. Recent work has shown that the most cytotoxic, oligomeric species of the E22G (arctic) variant of Aβ_1−42_ interacted more strongly with 1-anilinoaphthalene 8-sulfonate (ANS), a dye sensitive to exposed hydrophobic patches [112]. A higher degree of solvent-exposed, hydrophobic regions was further shown to lead to a disturbed cellular calcium homeostasis, likely due to disruption of the cell membrane [98]. Moreover, oligomers have been shown to bind with higher affinity and cause more disruption of synthetic membranes as compared to the higher-ordered fibrils [113]. These data emphasize the importance of intrinsic disorder and molecular flexibility of Aβ oligomers for the toxicity mechanism.

In conclusion, a re-evaluation of the oligomer cascade hypothesis is needed (reviewed in [114]). Whereas earlier hypotheses held one single oligomer of a predefined size responsible for toxicity [23, 115], it is obvious that a diverse “Aβ oligomeric soup” exists, consisting of a large variety of rapidly exchangeable polymorphs that differ in size, conformation, hydrophobicity, solvent exposure, intrinsic disorder (or internal flexibility), and toxicity. The oligomer cascade hypothesis should take into account that it is likely that the entire dynamic Aβ oligomeric soup contributes to the heterogeneity of AD progression and phenotype, via various toxic mechanisms.

## Intermolecular dynamics

As the in vivo Aβ pool is a mix of species influencing one another, one must also consider the dynamics between different Aβ species when regarding Aβ-related toxicity. First, Aβ peptides of various lengths are produced due to the heterogeneous cleavage pattern of APP by γ-secretase [5, 6]. This gives rise to the production of Aβ_1−40_, smaller amounts of Aβ_1−42_, and trace amounts of peptides ranging in length from 37 to 49 amino acids [116–118]. Second, a dynamical equilibrium exists between different aggregation states during Aβ aggregation. Studying the behavior of Aβ peptide mixtures and revealing the dynamics of interconversion among different aggregate species will be crucial in understanding the AD-related toxic effects of Aβ.

### The in vivo Aβ pool: a cocktail of different interacting species

The large majority of biophysical and cell biological studies investigating the role of Aβ in AD have focused either on pure Aβ_1−40_ or on pure Aβ_1−42_, the two predominant Aβ alloforms present in the brain [7, 119]. The in vivo Aβ pool not only contains different Aβ peptide lengths but also comprises post-translationally modified Aβ [120] (Fig. 1). Aβ peptides can undergo racemization [121, 122], isomerization [123], phosphorylation [124, 125], oxidation [126, 127], non-enzymatic glycation [128], and pyroglutamylation [129]. Post-translational oxidation of Met_35_ affects fibril flexibility within Aβ plaques [127]. Met_35_ oxidation also has been shown to impede the rate of Aβ aggregation in vitro [30], possibly by decreasing the β-strand content of the C-terminal region [130]. Furthermore, proteins can become modified by non-enzymatic glycation upon aging. Advanced glycation end products (AGEs), found in Aβ plaques and in neurons, and their receptor RAGE play an important role in AD by contributing to oxidative stress and by triggering inflammation signaling pathways [128, 131, 132]. For other modifications, it remains largely unknown how they can affect Aβ aggregation dynamics.

Various forms of Aβ co-exist and co-deposit in amyloid fibrils and plaques [23, 128]. It has become clear that biologically relevant mixtures of Aβ alloforms behave in a more complex manner in vitro than anticipated from their behavior in isolation, in terms of aggregation properties and toxicity [8–12]. For example, Aβ_1−38_ and Aβ_1−40_ exerted little toxicity in isolation, but were highly toxic to a neuroblastoma cell line when tested in a mixture, whereas addition of Aβ_1−38_ to Aβ_1−42_ had a protective effect [10].

Recently, it has been demonstrated that minor shifts in the Aβ_1−42_:Aβ_1−40_ ratio can modulate neurotoxicity [8]. The aggregation of samples of Aβ lengths in various compositions were monitored by NMR allowing simultaneous investigation of both Aβ_1−42_ and Aβ_1−40_ in the same sample by combining ^15^N-isotope-labeling of one Aβ alloform with ^15^N-edited filter experiments [9]. It was revealed that Aβ_1−42_ and Aβ_1−40_ directly interact and influence oligomer formation and aggregation kinetics. Moreover, cross-seeding data revealed structural differences between the different ratios at the level of the oligomeric state. A subtle change in the Aβ_1−42_:Aβ_1−40_ ratio was suggested to induce differences in conformational plasticity of the oligomeric peptide mixtures [9]. High molecular weight (HMW) mass spectra further showed that a continuous range of oligomeric intermediates were formed upon incubation of Aβ through a monomer addition process for the time frame within which toxicity exists [8, 9]. This observation is in agreement with the “coalescence and reorganization model of amyloid formation” [133], but also with the principle of a template-dependent dock-and-lock-and-block mechanism whereby the locking of a peptide cannot efficiently occur unless the previously loaded peptide has assembled into the correct position [134]. This can be envisaged in the following way: intrinsically disordered Aβ monomers diffuse freely and can attach individually to each other, to a pre-existing oligomer or to the fiber surface, especially through the distal ends. The crucial step occurs when the incoming monomer collides with the docking surface. In the case of a productive association, a permanent attachment can then take place, perhaps accompanied by a minor structural rearrangement. The conformational constraints of the monomers will therefore influence the efficiency and kinetics of the aggregation as well as the architecture of Aβ fibrils [135] (see “Aβ fibrils are polymorphic entities”). Alloform differences of the monomeric conformation are essential at this point to interpret productive or non-productive interactions [38, 40, 41], particularly in the complex in vivo pool of peptides. Aβ_1−42_ has the tendency to sample more fibril-like conformations compared to Aβ_1−40_ and as such can simply dock to fibril-like oligomers leading to highly productive (on-pathway) interactions. The more rigid and less flexible C-terminus of Aβ_1−42_ was suggested to enable the formation of a larger number of intramolecular contacts than Aβ_1−40_ [37, 40] and therefore provide a more extensive hydrophobic surface for intermolecular interactions. Experiments using amino acid substitutions in the C-terminal part of Aβ_1−40_ and Aβ_1−42_ confirmed that (i) the stability of the β-hairpin structure was increased by reducing the backbone flexibility and strengthening the hydrophobic interactions between the putative β-strands, (ii) destabilizing mutations in the C-terminal part of Aβ_1−42_ lead to a more Aβ_1−40_-like behavior, and (iii) stabilizing mutations in the C-terminus of Aβ_1−40_ lead to a more Aβ_1−42_-like behavior [143]. The conformational search of the incoming peptide for binding on the docking surface and for the proper orientation to lock-in-place could explain the complex aggregation behavior of Aβ alloform mixtures. The balance between productive and non-productive interactions in the transient encounter states is essential to guide the kinetics of aggregation, which in turn will define the time window within which the toxic species exist. Now that it becomes evident from independent research groups that the pattern of oligomer formation is mainly influenced by (patho)physiologically relevant Aβ_1−42_:Aβ_1−40_ ratios [136], it is also important to realize that independent (on- and off-) pathways exist for oligomerization and fibrillization of Aβ [137, 138].

### Experimental approaches to obtain insight into complex Aβ dynamics

It seems logical that the assembly and disassembly of toxic species is a dynamic and continuous process, at least in the initial stages, that is directed by the Aβ pool composition. However, the possibility that toxicity is present over a series of conformers or sizes should not be disregarded [91, 92, 94, 139, 140]. The question is thus how biophysical parameters influence this process in vivo and affect the relative distribution of Aβ species over toxic and non-toxic conformations over time. Given the complexity of the biophysical environment in which Aβ aggregation occurs in vivo, such a question is extremely difficult to address. Nevertheless, it is possible to analyze the dynamic features of this process in simplified and controlled conditions in vitro, and to evaluate the effect of the relative concentrations of Aβ_1−40_ and Aβ_1−42_ (and other alloforms) to the generation of neurotoxic species over time.

The combination of high-resolution NMR and HMW MS is perfectly suited to investigate the individual aggregation behavior of the diverse Aβ alloforms in complex and heterogeneous sample compositions. This can yield a comprehensive *aggregation fingerprint* that allows us to understand how the different compositions of the Aβ peptide pool influence the overall aggregation behavior. This aggregation fingerprint can be related to cytotoxicity, membrane integrity, apoptotic responses, and functional read-outs such as microelectrode arrays (MEA), in which synaptic activities at different timeframes and under various conditions are monitored in response to Aβ [8, 141]. Such a fingerprint also opens perspectives to the diagnostics and therapeutics field when it can be correlated to biomarkers. Patient-specific treatment (personalized medicine) could be based on the detailed characterization of the composition of the Aβ pool. It will be essential to correlate the aggregation fingerprint of such compositions with disease severity and the (ir)reversibility of the disease “progress”. It is also important to cover the overall dynamics in these pools rather than focusing on particular “toxic” intermediates that are only transient in the aggregation process. This will allow tackling the source of toxicity and limiting the time frame in which the toxic assemblies can exist. Aggregation fingerprints will thus be essential to better understand the Aβ-induced pathogenesis of AD and the biophysical processes that underlie the cell biological responses.

### The interaction between different species present during Aβ aggregation

NMR relaxation measurements showed that monomers are constantly binding to and being released from oligomers in vitro [142, 143]. Estimates showed that approximately 3 % of the peptide within the oligomer undergoes exchange with free monomer in pseudo-equilibrium conditions, suggesting that exchange occurs predominantly from the oligomer surface. A large part of the hydrophobic C-terminal region is involved in the association of monomer onto the oligomer surface [142]. In a next aggregation phase, protofibrils are formed that are also in constant exchange with monomers through the same surface region [144]. An elegant combination of ^19^F-NMR and other biophysical techniques revealed a heterogeneous mixture of small Aβ oligomers that exist in pseudo-equilibrium with protofibrils and fibrils during the early stages of aggregation [145].

Protofibrils self-associate and give rise to mature fibrils that can thermodynamically be considered as the most stable aggregation state due to the high density of intermolecular hydrogen bonding and steric zipper interactions [146]. However, fibrils are not static and irreversible end species, as was the traditional view, but were shown to continuously dissociate and reassociate through both fibril ends [147]. Aβ_1−40_ fibrils recycle to a greater extent than Aβ_1−42_ fibrils, which could be attributed to a difference in fibril dissolution rate. These findings are consistent with a dynamical model for interpreting plaque morphology, in which aggregation and disaggregation were proposed to be in steady-state equilibrium [148]. The species involved in the fibril recycling process are still a matter of debate. Differential solution NMR isotope labeling experiments revealed that Aβ_1−40_ monomers can replace Aβ_1−42_ on Aβ_1−42_ aggregates, recycling Aβ_1−42_ monomers back into solution [14]. Later reports confirmed the constant recycling of Aβ_1−40_ and Aβ_1−42_ monomers and competition of binding for the ends of protofibrillar and fibrillar aggregates [13]. Alternatively, the accumulation of fibrils could be associated with the generation of diffusible lower molecular weight aggregates. This idea is consistent with the observation of a halo of oligomeric Aβ surrounding senile plaques when analyzed by array tomography [149]. Recently, Knowles and coworkers demonstrated that the secondary nucleation pathway can be a major source of oligomers once the critical concentration of amyloid fibrils (in the order of 10 nM) has formed [150]. Hereby, the surfaces of existing fibrils catalyze the nucleation of new aggregates from the monomeric state, with a rate dependent on both the concentration of the monomers and that of the existing fibrils. As the critical fibril concentration is lower than the aggregate loads present in brains of AD patients, this pathway is likely to be active in the brain [150].

### The dynamical equilibrium potentially contributes to Aβ-associated toxicity

The co-existence of different Aβ aggregate species should be taken into account when analyzing Aβ toxicity studies. For example, fibrils act as a reservoir of soluble aggregates that can diffuse and induce toxic effects. The halo of oligomers surrounding senile plaques co-localizes with loss of excitatory synapses and spine collapse [149] and the disruption of dendritic spines in the vicinity of plaques is dependent on their distance from these plaques [151]. Moreover, fibrils can be destabilized by brain lipids and reverted into neurotoxic soluble protofibrils [139]. Amyloid fibrils can thus be toxic per se (see “Fibril polymorphism could lead to different pathological outcomes”) or can function as a potential source of neurotoxic oligomeric species [152, 153]. It has also been suggested that the ongoing polymerization process, rather than the formation of one stable aggregate, is responsible for Aβ-related toxicity [19, 154]. In accordance with this hypothesis, crude Aβ_1−42_ preparations containing a monomeric and heterogeneous mixture of Aβ_1−42_ oligomers and protofibrils were more toxic than purified monomeric, protofibrillar fractions or fibrils. The toxicity of protofibrils was directly linked with their interactions with monomeric Aβ_1−42_ and strongly dependent on their ability to convert into fibrils. Moreover, the ongoing Aβ aggregation process, rather than distinct aggregation states, elicited alterations in astrocyte metabolic phenotypes [19]. Therefore, insight into the dynamic equilibrium is required to fully understand Aβ toxicity.

## Other players in the game

The modulation of Aβ production, aggregation, and degradation by environmental factors [155–157], genetic risk factors [158–161], post-translational modifications [127], and an individual’s lifestyle [162–169] has been extensively reviewed before and does not lie in the scope of this review. Only a few reports discuss the influence of these factors on Aβ dynamics.

Metals have been shown to affect Aβ intramolecular dynamics. Binding of zinc to the N-terminus of the Aβ monomer leads to a decrease in the intrinsic mobility of this region and the formation of a turn-like conformation in residues Val24-Lys28 promoting aggregation, as shown by ^15^N relaxation measurements [170]. Copper can also bind to the N-terminus, causing a structural ordering in this region [171], but slowing down aggregation [110].

There is evidence that membrane composition and properties, in turn, play a critical role in Aβ cytotoxicity associated with its conformational changes and aggregation into oligomers and fibrils ([172–174], reviewed in [175]). Moreover, interaction with lipid membranes can modulate Aβ peptide conformation and aggregation properties (reviewed in [175, 176]).

Genetic evidence suggested a role for chaperones in AD [177] and abundant chaperone levels block formation of Aβ aggregates as was demonstrated in a *Caenorhabditis elegans* disease model [178]. In vitro results indicated a role for heat shock proteins in the early aggregation events by interfering with the dynamical aggregation process [179]. The BRICHOS domain, a chaperone-like domain found in lung surfactant protein C, is reported to be a potent in vitro inhibitor of Aβ aggregation [180]. The contribution of chaperones in the context of AD is reviewed in [181].

Interactions of Aβ with small molecules designed to target Aβ toxicity and/or Aβ aggregation have also been extensively studied. These ligands are not only interesting in light of drug development, but also provide a tool for addressing the modulation of Aβ dynamics upon ligand interaction [182–184].

As the Aβ monomer concentration affects the dynamical equilibrium between monomers, oligomers, protofibrils, and fibrillar Aβ, it is also worthwhile to consider factors that modulate Aβ metabolism. Aluminium is known to increase the Aβ brain burden in experimental animals and this might be due to a direct influence upon Aβ anabolism or to direct or indirect effects on Aβ catabolism [185]. Holtzman and coworkers reported that human cerebrospinal fluid Aβ levels undergo diurnal fluctuations and that this cycle is disturbed following plaque formation before the appearance of any cognitive symptoms [186]. Aβ fluctuations were affected by perturbation of the orexin signaling pathway and the sleep-wake cycle and this suggested that sleep abnormalities in earlier life might predispose an individual to AD [187]. Cholesterol has been suggested to provide stability to membrane-adjacent lipid rafts and therefore facilitate the Aβ cleavage from APP [168]. Recent evidence showed that the γ-secretase subunit composition defines the Aβ profile and affects the ratio between alloforms [6]. This implies that external factors influencing the γ-secretase subunit composition will have a profound effect on Aβ toxicity.

## Conclusions

Understanding the intrinsic molecular flexibility, dynamics of interactions, and the structural behavior of the various Aβ peptides is crucial to comprehend the molecular mechanisms underlying the pathophysiology of Alzheimer’s disease. This will allow a more rational design of therapeutic intervention strategies to halt the disease progress and neutralize the malignant action of Aβ aggregation. To gain understanding of these events is difficult if not impossible to follow in real-time in the human brain. Therefore, these events are often mimicked in the test tube in research laboratories where information on Aβ behavior can be followed in molecular detail using advanced biophysical and biochemical assays in the course of seconds to hours or days, which happen in patients over a range of years.

The intrinsically disordered nature of Aβ raises the question of whether this peptide may act as signaling peptide, which is known to require a high degree of flexibility. It is striking to observe that many proteins involved in human diseases are in fact classified as IDPs (alpha-synuclein, tau, multiple prions) [188, 189]. This raises the question as to whether protein flexibility may act as a disease-contributing factor as opposed to the generally accepted idea that specific sizes or conformations of oligomeric forms of these peptides induce pathogenesis. In this review we state that different types of dynamics can be distinguished varying from inter- to intramolecular factors as well as external factors and that recent observations strongly indicate that indeed the contribution of dynamics to pathogenesis warrants further investigation. As the dynamic nature of Aβ and its ability to undergo conformational changes and aggregation has hampered its study, promising new experimental approaches and chemical tools [182] are being developed to address Aβ dynamics, having the major advantage that they can be used directly without the need for modification of Aβ with additional amino acids or fluorophores [110, 190]. While a lot has been learned in the past from the behavior of the Aβ system, it is clear that the picture is still incomplete and extremely complex. Variability in terms of space (intra- and extracellular space, brain compartments, patient-to-patient differences, etc.) and time (circadian rhythm, aging, lifestyle, etc.) imposes additional dynamical factors, emphasizing the importance to better understand the fluctuating microenvironment. Therefore, it is opportune to compare the Aβ system to a complex ecosystem or society, where minor perturbations might have profound effects that can result in cataclysmic events. Various Aβ alloforms interact and mutually influence each other’s behavior, but they also interact with the complex biological cell surface where they might exert a toxic effect by interfering with its normal functionality. Therefore, a holistic view of the dynamical Aβ ecosystem would enable us to initiate a successful ecosystem management strategy to prevent or remediate the AD pathobiology.

We summarized the evidence supporting the role of structural flexibility and in particular of the intrinsic protein disorder in the Aβ system to AD pathogenesis. A more systematic approach to the study of molecular flexibility in the Aβ system is required. This knowledge should then be integrated into future research efforts to optimize the clinical outcomes of drug trials.
